# Nanocarriers in Tuberculosis Treatment: Challenges and Delivery Strategies

**DOI:** 10.3390/ph16101360

**Published:** 2023-09-26

**Authors:** Mahesh Kumar, Tarun Virmani, Girish Kumar, Rohitas Deshmukh, Ashwani Sharma, Sofia Duarte, Pedro Brandão, Pedro Fonte

**Affiliations:** 1 School of Pharmaceutical Sciences, Modern Vidya Niketan University, Palwal 121105, India; sorotmahesh@gmail.com (M.K.); girish.kumar@mvn.edu.in (G.K.); ashwani.pharmacy@mvn.edu.in (A.S.); 2 Institute of Pharmaceutical Research, GLA University, Mathura 281406, India; rahi18rahi@gmail.com; 3 iBB—Institute for Bioengineering and Biosciences, Department of Bioengineering, Instituto Superior Técnico, University of Lisboa, 1049-001 Lisbon, Portugal; sofia.duarte@tecnico.ulisboa.pt (S.D.); pbrandao@egasmoniz.edu.pt (P.B.); 4 Associate Laboratory i4HB—Institute for Health and Bio-Economy, Instituto Superior Técnico, University of Lisboa, Av. Rovisco Pais, 1049-001 Lisbon, Portugal; 5 Egas Moniz Center for Interdisciplinary Research (CiiEM), Egas Moniz School of Health & Science, 2829-511 Almada, Portugal; 6 CQC-IMS, Department of Chemistry, University of Coimbra, Rua Larga, 3004-535 Coimbra, Portugal; 7 Center for Marine Sciences (CCMar), University of Algarve, Gambelas Campus, 8005-139 Faro, Portugal; 8 Department of Chemistry and Pharmacy, Faculty of Sciences and Technology, University of Algarve, Gambelas Campus, 8005-139 Faro, Portugal

**Keywords:** tuberculosis, ligand, nanoformulation, nanoparticle, patent, mannose, folic acid

## Abstract

The World Health Organization identifies tuberculosis (TB), caused by *Mycobacterium tuberculosis*, as a leading infectious killer. Although conventional treatments for TB exist, they come with challenges such as a heavy pill regimen, prolonged treatment duration, and a strict schedule, leading to multidrug-resistant (MDR) and extensively drug-resistant (XDR) strains. The rise of MDR strains endangers future TB control. Despite these concerns, the hunt for an efficient treatment continues. One breakthrough has been the use of nanotechnology in medicines, presenting a novel approach for TB treatment. Nanocarriers, such as lipid nanoparticles, nanosuspensions, liposomes, and polymeric micelles, facilitate targeted delivery of anti-TB drugs. The benefits of nanocarriers include reduced drug doses, fewer side effects, improved drug solubility, better bioavailability, and improved patient compliance, speeding up recovery. Additionally, nanocarriers can be made even more targeted by linking them with ligands such as mannose or hyaluronic acid. This review explores these innovative TB treatments, including studies on nanocarriers containing anti-TB drugs and related patents.

## 1. Introduction

Among the most important global health challenges are infectious diseases such as tuberculosis (TB), acquired immunodeficiency syndrome, and human immunodeficiency virus infection [1,2]. *Mycobacterium tuberculosis*, an aerobic, Gram-positive, non-motile, acid-fast tubercular, rod-shaped bacillus, causes airborne TB, which mostly affects the lungs but may also impact extra-pulmonary regions [3]. Due to their lipid-rich cell walls, mycobacteria may live within alveolar macrophages [4]. The tubercle bacillus, *M. tuberculosis*, which is spread via airborne droplets and can remain, live, and divide every 16–20 h inside alveolar macrophages, is the principal method of transmission for this dangerous illness [5]. Per the latest report by World Health Organization (WHO), about 10.6 million cases of TB were reported in 2021, comprising 6 million men, 1.2 million children, and 3.4 million women [6]. It was estimated that about 1.6 million people died from TB in 2021 throughout the world. About 80% people infected by TB reside in low- and middle-income countries. The main causes for TB include weakened immune system, chewing of tobacco, undernourishment, and other complications such as diabetes and HIV infection [6]. In 2021, 2.2 million new TB cases were attributed to undernourishment, 740,000 to alcohol use disorders, and 690,000 to smoking throughout the globe. To reach the global goal set at a high-level UN meeting on TB in 2018, USD 13 billion is required annually for TB prevention, diagnosis, treatment, and care. It is expected that TB detection and treatment saved 74 million lives between 2020 and 2021 [7]. TB is the second highest infectious cause of death after COVID-19 and the thirteenth major cause of mortality across the world [8]. TB exists in all nations and among all age groups, but it can be treated and avoided. One of the Sustainable Development Goals (SDGs) of the United Nations is to end the TB epidemic by 2030 [9].

Chemotherapy is currently the only option for the clinical management of TB patients, with cure rates of up to 95% when given correctly to those with drug-susceptible TB. However, the majority of anti-TB medicines have subpar pharmacokinetic characteristics, which frequently prevent them from performing to their full potential in clinical situations [10]. Poor bioavailability due to variable drug absorption and unwanted first-pass metabolism, lengthy regimens with high dosing frequencies, and individual and combined drug toxicity as well as severe adverse effects are some of the issues related to the therapeutic limitations of the current anti-TB regimens. These challenges contribute to low patient adherence, therapeutic failure, and the alarming emergence of multidrug-resistant (MDR) strains, all of which explain TB’s current lethal state and the pressing need to advance anti-TB treatment [11,12].

Advanced drug delivery systems require the development of a nanotechnological technique, which is a rapidly evolving cutting-edge scientific field that includes a wide range of disciplines such as chemistry, physics, and biology as well as special nanodimension structures with therapeutic applications in pharmacology and the biomedical field [13]. Many researchers are interested in the development and standardization of nanocarriers for various reasons, such as reduction in drug doses, minimal adverse effects, solubility and bioavailability improvement of drugs, targeted drug delivery resulting in improved patient compliance, and acceleration in recovery of patients [14,15]. These nanocarriers include solid lipid nanoparticles, nanostructured lipid carriers, liposomes, nano-emulsion, nanosuspension, nanoparticles, polymeric micelles, and dendrimers [16]. The nanocarriers appear to be a viable and intriguing approach to solve the limitations associated with conventional treatment associated with TB [17].

Besides the above mentioned treatment, vaccination is also another option for prevention of TB [18]. The WHO advises that, despite the Bacille Calmette–Guerin (BCG) vaccine’s success in preventing TB and reducing mortality among infants and young children who have received vaccinations since birth, it is important to take into account the vaccine’s capacity to produce “trained immunity” by causing non-specific immune sensitization to other pathogens [19]. Additionally, it might aid in lowering the prevalence of infectious diseases, such as malaria, that are resistant to antimicrobials. Several benefits, including the large surface area of the sub-micron-sized particles, increased interaction of the vaccine with the large surface area of the respiratory mucosa and enhanced penetration into bacilli-loaded granulomas attributed to nanotechnology-based approaches, may particularly benefit in targeting the most common respiratory forms of TB against which BCG appears to be ineffective [20]. Previous research has shown the ability of the antigens implanted on nano-particulate platforms to improve immune response to other pathogens causing other infectious diseases, indicating the possibility of TB vaccines having the same capability once created [21].

## 2. TB Pathophysiology

**Figure 1:**
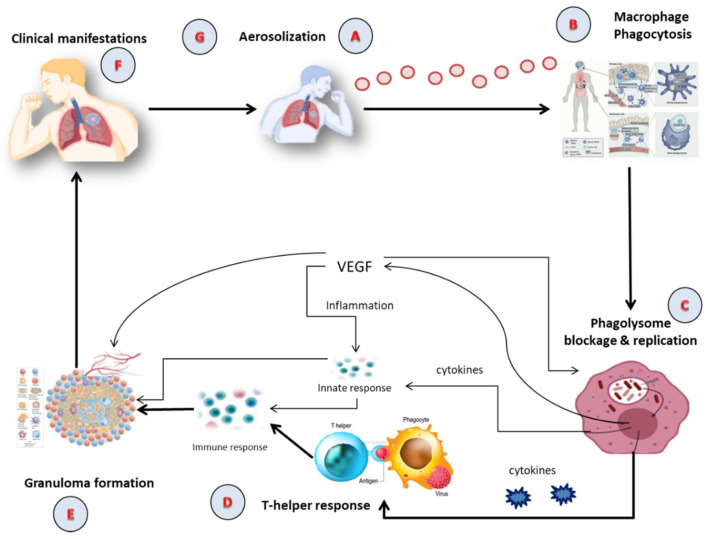
The Pathophysiology of active TB; (**A**) Aerosolization; (**B**) Macrophage phagocytosis; (**C**) Blockage and replication of phagolysosome; (**D**) T-helper response; (**E**) Granuloma formation; (**F**) Clinical manifestation; (**G**) Infection of susceptible person via aerosolization. VEGF: Vascular Endothelial Growth Factor.

Tubercle bacilli nuclei in droplets that reach the lungs’ alveoli during breathing cause infection, in a step called aerosolization (Figure 1A) [22,23]. These tubercle bacilli are ingested by alveolar macrophages, the majority of which are killed or inhibited (Figure 1B) [24]. After preventing the acquisition of the phagosome and lysosome, *M. tuberculosis* reproduces intracellularly inside the macrophages (Figure 1C). Asymmetric cell division is a special kind of cell division seen in *M. tuberculosis* [25,26]. Those bacteria may spread, if they are alive, through the lymphatic system or the circulation to the regional lymph nodes, the apex of the lung, kidneys, brain, and bony parts of the body, where TB sickness is most likely to develop. This process of dissemination sets the immune system for an expanded response (Figure 1D) [27]. To use an analogy, a bacterial jail called a granuloma aims to isolate a bacterium beneath an enclosure of immune cells. Both macrophages and lymphocytes that surround and enclose *M. tuberculosis* constitute the granuloma itself. TH1, natural killer (NK) cells, dendritic cells, macrophage, regulatory T cells (Treg), foam cells, giant cells, epithelioid macrophage, neutrophils, and B cells are some of the cells implicated in the granuloma (Figure 1E). In clinical significance, primary and secondary TB are the two forms of TB (Figure 1F). In immunocompromised individuals, primary infection is the one that develops when the immune system cannot handle it. At this point (Figure 1G), the infected person releases infectious aerosols of *M. tuberculosis* and infects the next susceptible person. Suppose *M. tuberculosis* is present but not eradicated by the immune system or granuloma. In such instances, the illness is believed to be latent and could turn into secondary TB.

The intricate nature of TB pathophysiology underscores the necessity for precise and effective diagnostic methods. As we delve into the pathogenesis of TB, understanding how tubercle bacilli operate within the human body, from aerosolization in the lungs’ alveoli to the formation of granulomas and eventual bacterial spread, we can appreciate the challenges faced in diagnosis. The ways in which *M. tuberculosis* interacts with our immune system, spreads, and manifests form a complex web of biological interactions, making its detection a critical task. Moving from understanding these interactions to their clinical implications, it becomes evident why varied diagnostic methods have emerged.

## 3. TB Diagnostic

**Figure 2:**
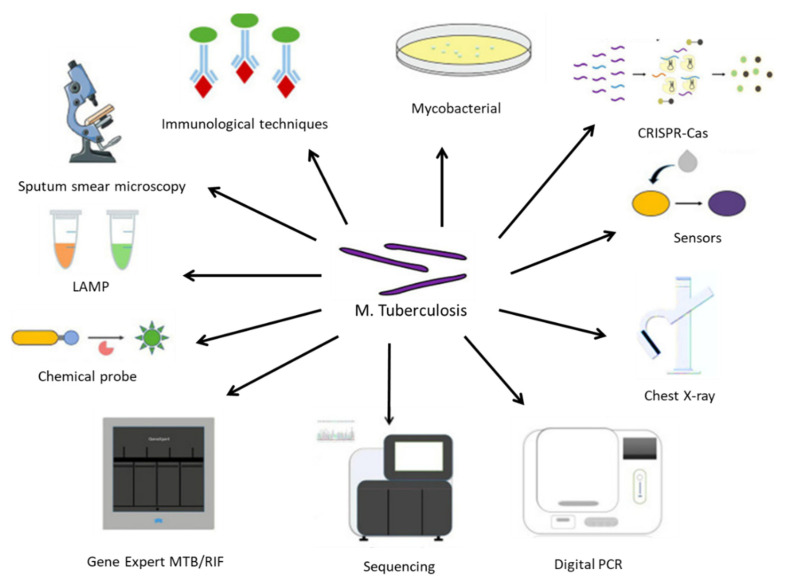
An array of diagnostic instruments for point-of-care testing for TB. LAMP: Loop-mediated isothermal amplification; MTB: *Mycobacterium tuberculosis* complex; RIF: resistance to rifampin. Reprinted with permission from [34].

There are several ways TB can be diagnosed, as summarized in Figure 2. The different methods display different advantages and disadvantages. For example, the sputum smear microscopy (SSM) examination remains one of the most accessible and affordable diagnosis tools and is often the only available technique in developing countries. However, its poor sensitivity and high rate of false negatives can lead to misdiagnosis or under-diagnosis and delay the start of an effective therapeutic approach. Some recent upgrades to this technique, involving the use of fluorescent antibodies or digital pathology tools, might help to overcome some of its current limitations, but they also increase costs and might not constitute a desirable approach in countries with deficient healthcare systems [28]. Another gold standard approach is the culture of *M. tuberculosis* in specific growth media, which often can simultaneously allow for the evaluation of antibiotic susceptibility [29].

Molecular biology tools include Xpert RIF/MTB, which reduces the time required for a diagnosis while improving its sensitivity; loop-mediated isothermal amplification (LAMP) [30]; and droplet digital polymerase chain reaction (ddPCR), which enables accurate diagnosis even with very small amounts of contaminated samples [31].

Immunoassays are often valuable when the collection of infected secretions might be challenging, such as in pediatrics or in patients with mild symptomatology. The tuberculin skin test or interferon-ƴ release assay are often used. However, they do not distinguish between an active infection and a vaccination-induced immunological response, and they are often unreactive in immune-compromised individuals [32]. Immuno-PCR is another alternative that enables the detection of circulating antibodies and/or mycobacterial antigens in blood samples or other fluids from patients [33].

With methodological advances based on increasingly sensitive equipment (such as flow cytometers, matrix-assisted laser desorption ionization-time of flight (MALDI-TOF) spectroscopy), chemical probes, immunosensors, and next-generation sequencing tools, it is expected that the diagnosis of *M. tuberculosis* infection will be an easier task, which hopefully will result in more appropriate therapeutic outcomes and decrease the socioeconomic burden of this disease [34].

## 4. Conventional Treatment Options and Its Limitations

The current vaccination used to prevent TB is called *Mycobacterium bovis* bacillus Calmette–Guérin (BCG), and it was first given out in 1921 [35]. The BCG vaccine protects against TB in children between 60 and 80 percent of the time, but it is ineffective against pulmonary TB in adults [36]. Instead of the BCG vaccine, which is only effective in children, the WHO advises pharmacological therapies for TB in adults; their efficacy has also been reported to differ geographically [37]. Furthermore, because BCG is a live vaccine, those with impaired immune systems may acquire a disseminated infection. There is a pressing need to create new vaccines due to these limitations [38].

**Figure 3:**
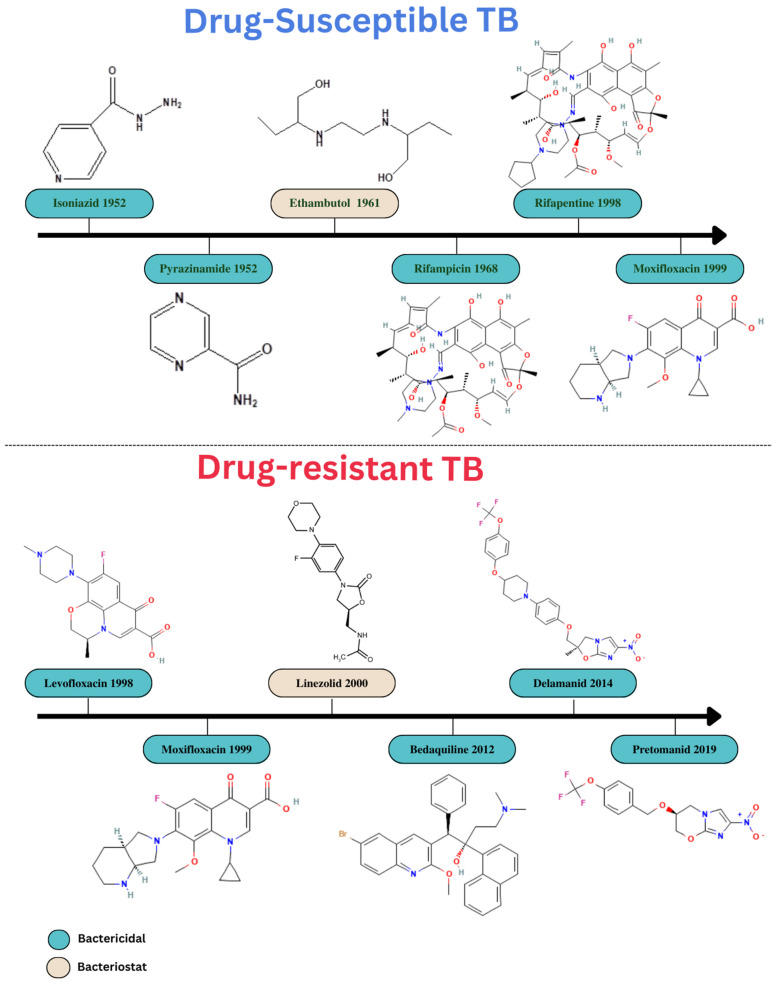
Progression in drug development for treatment of TB.

In most countries, some drug-susceptible TB is treated with an oral medication regimen. This treatment plan consists of two months of daily or three-times-per-week administration followed by four months of isoniazid and rifampicin [39] of ethambutol (E), isoniazid (H), pyrazinamide (Z), rifampicin (R), and HRZE [40]. Treatment regimens have become increasingly complex due to the rise in TB complications, namely MDR strains, extensively drug-resistant (XDR) strains, HIV co-infection, the presence of comorbidities such as diabetes, and TB retreatment after recurrence. Drug-resistant TB may be treated by prolonging the course of treatment with drugs that the organism is susceptible to for up to 20 months or by adding an injectable antibiotic such as kanamycin, amikacin, or streptomycin [41]. The progression of anti-TB medications that have been authorized and are used for treatment of TB has been depicted in the Figure 3.

The currently available drugs for treatment of TB are distributed throughout the body via systemic blood circulation after being ingested or administered intravenously, and many molecules aggregate in other body regions rather than reaching the intended site, leading to adverse effects such as nephrotoxicity, hepatotoxicity, ocular toxicity, and ototoxicity. The majority of anti-TB medications are taken orally, which causes pharmacokinetic problems such as reduced bioavailability and a low therapeutic index [20]. Drug therapy used in the traditional manner requires a protracted therapy regimen that involves the continuous and frequent administration of several medications, which lowers patient adherence to current therapies. This is a crucial marker of infection recurrence, as well as the development of both the more serious XDR-TB and MDR-TB [11].

In addition, patients are less likely to adhere to existing medications as a result of time duration concerns in conventional pharmacological therapies, which accounts for infectious recurrence and the emergence of MDR and XDR-TB. MDR-TB is a growing problem for the healthcare sector in developing countries [42]. Although current anti-TB therapies are effective, it is urgently necessary to develop new short-course regimens with additional drugs to address the various challenges associated with drug and target selection as well as patient commitment. Additionally, contemporary strategies such as nanoparticles, liposomes, dendrimers, etc. must be developed to address the conventional therapeutic challenges.

## 5. Nano-Based Drug Delivery in TB

Due to various benefits such as lower doses, improved dosage regimens, reduced adverse effects, decreased drug degradation, improved solubility and bioavailability, and improved patient compliance over conventional therapy, nanomedicines have been shown to be effective therapies and result in encouraging outcomes for the treatment of TB [43,44,45,46]. A wide variety of drug delivery systems using different types of nanocarriers have proven to be successful [45]. Controlled and sustained drug release is one advantage of nanocarrier-based anti-TB medications over free medicines [47]. They also decrease dosing frequency and address the issue of poor compliance [16]. Physical encapsulation, adsorption, or chemical conjugation are all ways that therapeutic drugs can be introduced into nanocarriers. Significantly, it is possible to target host cells utilizing nanocarriers via either passive accumulation or active targeting [48].

Nanosized drug delivery systems advantages can be summarized as follows:

- Targeted Drug Delivery: Nanocarriers can deliver drugs directly to the infected site, ensuring that the therapeutic agents reach the desired location in the body, thus increasing the efficacy and reducing potential side effects [48].
- Enhanced Bioavailability: Nanoformulations can increase the solubility of poorly water-soluble anti-TB drugs, leading to better absorption and higher bioavailability [16,43].
- Reduced Drug Dosage: Due to their efficient delivery and release mechanisms, nanosized drug delivery systems can often achieve therapeutic effects with reduced drug doses, minimizing potential side effects and toxicity [16].
- Overcoming Drug Resistance: Nanocarriers can be engineered to counter multidrug-resistant TB strains by co-delivering multiple drugs or by protecting the drug from degradation [43,44].
- Controlled Release: The drugs encapsulated in nanocarriers can be released in a sustained manner over time, ensuring consistent drug levels and potentially reducing the frequency of dosing [47].
- Reduced Side Effects: Targeted delivery and reduced dosage mean that healthy tissues are less exposed to the drug, which can minimize side effects [43,44].
- Improved Patient Compliance: Nanosized drug delivery systems can offer simplified dosing regimens, leading to better patient adherence to the treatment, especially considering the long treatment courses required for TB [43,44].
- Penetration of Biological Barriers: Nanocarriers can be designed to penetrate tough biological barriers, such as the blood–brain barrier, enabling treatment of TB manifestations in difficult-to-reach sites [43,44].
- Protection from Degradation: Some nanocarriers can protect the encapsulated drugs from enzymatic or pH-mediated degradation in the body, ensuring the drug remains active longer [43,44].
- Versatility: Nanosized delivery systems can be adapted or modified to carry various types of drugs, including small molecules, proteins, or even nucleic acids, offering flexibility in treatment strategies [43,44].
- Co-delivery of Multiple Drugs: Certain nanocarriers can encapsulate multiple anti-TB agents, allowing for combination therapy, which is often more effective and can reduce the likelihood of developing drug resistance [43,44].

By harnessing these advantages, nanosized drug delivery systems hold potential to revolutionize TB treatment strategies, addressing many of the challenges posed by conventional therapy.

### 5.1. Polymeric Nanoparticles (PNPs)

PNPs are appealing nanocarriers to increase the effectiveness of chemotherapeutics and reduce the toxic effects of anti-TB drugs by encapsulating and conjugating the therapeutic drugs [49]. The delivery of anti-TB drugs via encapsulation in PNPs improves the efficacy and proficiency of TB treatment due to their possession of various characteristics of improved bioavailability, reduced dose frequency, smaller size, greater drug loading capacity, improved stability, higher surface volume ratio, biocompatibility, biodegradability, and ease of modification [50,51]. PNPs provide reduced systemic toxicity owing to direct delivery of a drug to a specific site, which allows for minimal exposure of other organs to the drug. PNPs enable the reduction of drug resistance by enhancing intracellular uptake and concentration of the drug at the infected site [51]. In addition, PNPs enable the co-delivery of various drugs, which is a prime requirement of TB treatment because various drugs are administered simultaneously in TB treatment to avoid drug resistance [52]. PNPs can be functionalized to target the infected cells, which increases the application of PNPs in delivery of anti-TB drugs.

PNPs are composed of natural and synthetic polymers [53]. Albumin, collagen, chitosan, hemoglobin, and alginate are examples of natural polymers; however, they are not frequently used due to their high cost or low purity [54]. Synthetic polymers which are biodegradable, biocompatible, and stable include poly(amides), poly(amino acids), poly(alk-l-cyanoacrylates), poly(esters), and poly(orthoesters) [55]. PLGA (poly(lactide-co-glycolide)) copolymers are among those mentioned above that are frequently employed in anti-TB drug delivery. The primary benefit of these polymers is that they may break down inside the body via metabolic pathways and eliminated. PLGA has also been authorized for use in a number of medical applications. To prevent the entry of antibiotics and protein medicines, degradable PLGA polymers have been utilized [20]. These nanoparticles can be successfully phagocytosed by human alveolar macrophages, which causes a buildup of the drug in the intracellular milieu and results in bacterial oblation [56].

Pandey et al. [57] encapsulated three anti-TB drugs (isoniazid, rifampicin, and pyrazinamide) within PLGA nanoparticles (NPs) using the multiple emulsion method, which was followed by vacuum drying. These NPs were then administered to experimental animals via nebulization. The pharmacokinetics of each encapsulated drug was evaluated as well as the chemotherapeutic potential of the formulation in animals infected with *M. tuberculosis*. The majority of the NPs were found to have a particle size ranging from 186 to 290 nm with a polydispersity index of 0.38 ± 0.04. The entrapment efficiencies were 56.9 ± 2.7% for rifampicin, 66.3 ± 5.8% for isoniazid, and 68 ± 5.6% for pyrazinamide. The mass median aerodynamic diameter (MMAD) of the aerosolized particles was found to be 1.88 ± 0.11 µm, suggesting their suitability for pulmonary delivery. NPs loaded with rifampicin exhibited a sustained drug release in plasma over 6 days, whereas those loaded with isoniazid and pyrazinamide did so for 8 days. In contrast, the plasma levels of these drugs were detectable for only 12–24 h following oral or aerosol administration and for 6–10 h after intravenous administration. The C_max_ for NPs loaded with rifampicin and pyrazinamide was similar to that of oral administration of the respective standalone drugs. However, the C_max_ for isoniazid-loaded NPs was higher than its oral counterpart. The T_max_ for the drug-loaded nanoparticles was 24 h for those loaded with rifampicin and 96 h for the isoniazid and pyrazinamide ones. The elimination half-lives for the rifampicin, isoniazid, and pyrazinamide-loaded NPs were found to be 69.30 ± 4.00 h, 23.10 ± 2.00 h, and 69.00 ± 4.80 h, respectively. These values demonstrated a significant increase compared to both oral and intravenous administration of the parent drugs. The NPs encapsulating rifampicin, isoniazid, and pyrazinamide showed enhanced absolute bioavailability values of 6.50, 19.10, and 13.40, respectively, compared to both oral and intravenous dosing. Notably, upon nebulizing the drug-encapsulated NPs for five doses, no tubercle bacilli were detected in the lungs of guinea pigs infected with *M. tuberculosis*. In contrast, achieving a similar therapeutic effect required 46 daily doses using oral medication [57].

### 5.2. Solid Lipid Nanoparticles (SLNs)

Lipid nanoparticles have received a great deal of attention from researchers because they are at the cutting edge of the rapidly developing field of nanotechnology and show tremendous promise for reaching the goal of regulated and targeted medication delivery in the treatment of various kinds of infectious diseases [58,59,60]. SLNs offer a number of noteworthy advantages, including increased solubility, reduced side effects, increased bioavailability of pharmaceuticals, adaptation of encapsulation of both hydrophilic and hydrophobic medications, better stability, higher specificity, and increased likelihood of large-scale manufacture [16]. SLNs have similar properties to PNPs, but the main point is that they possess a better safety profile [61].

The more beneficial properties of SLNs are their size (less than 400 nm), simplicity of functionalization, chemical and mechanical stability, and improved transport of lipophilic therapeutic drugs [62]. Additionally, SLNs are able to penetrate many physiological barriers that prevent drugs from reaching infected sites and can bypass the multidrug resistance mechanisms that are common to TB treatment [63]. Due to their increased permeability and long retention times, SLNs have the unique inherent ability to concentrate the drug with precision at the infected site.

These are made up of either solid lipids or a combination of lipids and surfactants [64]. They may also contain an aqueous phase, surface modifiers, co-surfactants, stealthing agents, and cryoprotective chemicals [65]. A hydrophobic medication, or combination of hydrophobic drugs, is confined within the solid lipid matrix of SLNs, providing physical stability by shielding the molecule from chemical deterioration. These enhance the half-life of medications in blood circulation and alter their release pattern, increasing the therapeutic potency of the drugs [66].

Khatak et al. [67] fabricated SLNs containing three anti-TB drugs (rifampicin, isoniazid, and pyrazinamide) using stearic acid, compritol 888, poloxamer 188, and sodium taurocholate via the micro-emulsion method, intended for oral administration to target *Mycobacterium marinum*. The optimized SLNs, which contained stearic acid (X1) at 2% *w*/*w*, compritol 888 (X2) at 3% *w*/*w*, and a combination of poloxamer and sodium taurocholate (X3) at 3% *w*/*w*, exhibited a mean diameter of 187.9 ± 10.73 nm and a zeta potential of −47.4 mV. The entrapment efficiencies for rifampicin, isoniazid, and pyrazinamide were found to be 86.40 ± 0.274%, 83.84 ± 0.269%, and 81.43 ± 0.576%, respectively. The cumulative drug release from the drug-loaded SLNs in the first hour was 9.17%, 7.70%, and 10.26% for rifampicin, isoniazid, and pyrazinamide, respectively, in 0.1 N HCl, and 6.04%, 11.54%, and 10.34%, respectively, in phosphate buffer pH 6.8. In contrast, for the marketed tablet (Rifater), the initial 1-h release was 63.76%, 66.86%, and 65.35% for rifampicin, isoniazid, and pyrazinamide, respectively, in 0.1 N HCl, and 70.37%, 74.23%, and 71.37%, respectively, in phosphate buffer pH 6.8. The minimum inhibitory concentrations for the SLN-loaded rifampicin, isoniazid, and pyrazinamide were 2.16 µg/mL, 2.55 µg/mL, and 5.04 µg/mL, respectively. In comparison, the values for the standalone rifampicin, isoniazid, and pyrazinamide drugs were 6.25 µg/mL, 12.5 µg/mL, and 6.23 µg/mL, respectively, indicating the SLNs required lower drug concentrations. The IC50 value for the drug-loaded SLN was 0.9492, while the values for rifampicin, isoniazid, and pyrazinamide were 1.664, 1.818, and 1.224, respectively, indicating the enhanced toxicity potential of the drug-loaded SLN [67].

Obinu et al. [68] fabricated SLNs containing a novel drug, SS13, using two lipids, Witepsol and Gelucire, via a modified solvent emulsification–evaporation technique intended for oral administration. SLNs made with Gelucire (SLN-G) had a mean diameter of 247.1 ± 19.8 nm, a polydispersity index (PDI) of 0.772, and a zeta potential of −13.82 ± 2.44 mV. In contrast, SLNs created using Witepsol (SLN-W) had a mean diameter of 450.6 ± 14.9 nm, a PDI of 0.141 ± 0.030, and a zeta potential of −10.52 ± 2.33 mV. Both formulations had a mean diameter below 500 nm, suggesting suitability for intestinal absorption. The SLN-G and SLN-W treatments exhibited an initial burst release of 24% and 11%, respectively. After this, there was no further drug release from the formulations for up to 24 h, whereas the plain drug showed a 35% release in the first 2 h, followed by a reduced release rate, reaching 45% at 24 h. The apparent permeability coefficient for SLN-G and SLN-W was 8.23 × 10^−5^ and 8.61 × 10^−5^ cm/s, respectively, while for the pure drug it was 4.57 × 10^−8^ cm/s. This suggests that SS13-loaded SLNs offer enhanced intestinal mucosal permeability and increased absorption. MTT assay results indicated that all SLNs demonstrated improved biocompatibility at all treatment concentrations, except for the unloaded SLN with Gelucire (SLN-G) and SLN-G, which increased cell death at a 3 µM concentration. All SLNs showed a significant reduction in cell viability up to 1 µM concentration, leading to increased cell death as the dosage increased. The researchers concluded that SS13 could be incorporated into SLNs as a novel approach to treat multidrug-resistant TB [68].

Although SLNs provide several benefits in the treatment of cancer, they also have certain drawbacks, including low drug loading capacity, drug ejection, a higher incidence of polymorphic transitions, and unpredictable agglomeration, which must be addressed [69].

### 5.3. Nanostructured Lipid Carriers (NLCs)

NLCs have demonstrated their efficacy as advanced drug carriers in the treatment of infectious diseases to address a number of the aforementioned shortcomings of SLNs [70,71]. Due to their unique qualities, which include improved drug encapsulation, long-term chemical and physical stability of the encapsulated drug, surface changes, and site-specific targeting, they have a wide range of applications as drug carriers [72,73]. Since they combine liquid and solid lipids, their crystallinity is decreased, and their matrix system is loosely packed. Due to the overall improvement in drug entrapment capability and higher stability, further research is required [73].

Ahalwat et al. [74] developed NLCs for isoniazid. Their work highlighted the successful creation of a lipid matrix designed for pulmonary administration, aiming for an enhanced 24-h sustained release effect. This could potentially reduce drug dosage and modify dosing schedules. The optimized formulation showed a particle size, entrapment efficiency, drug loading, zeta potential, and PDI of 306.4 ± 3.53 nm, 72.39 ± 1.39%, 15.93 ± 0.30%, +19.08 ± 1.73 mV, and 0.539 ± 0.06, respectively. In vitro experimentation revealed a cumulative drug release of 66.35 ± 1.44%. The encapsulation of the drug in NLCs was validated via transmission electron microscopy. The optimized formulation was found to be stable under all storage conditions according to ICH guidelines [74].

Patil et al. [75] formulated clofazimine-entrapped NLCs and also developed clofazimine-entrapped mannosylated NLCs to target alveolar macrophages. The safety of both the drug-loaded NLCs and the drug-loaded mannosylated NLCs was assessed using cell viability studies, in vitro biocompatibility tests, and in vivo acute inhalation toxicity evaluations. The NLCs were found to be safer than drug dispersion in concentrations ranging from 2.5 to 25 g/mL. In acute in vivo toxicity testing, there were no observed behavioral or physiological changes, and no mortalities over a 14-day period. Hemocompatibility tests reported normal RBC count and minimal hemolysis of 0.23 ± 0.081% in the group treated with drug-entrapped mannosylated NLCs. Pharmacokinetic studies demonstrated that the sustained release of the drug, combined with mannose receptor-mediated endocytosis, achieved a maximum concentration (C_max_) of 35.44 ± 0.34 µg/g from the mannosylated-drug-NLCs after 48 h, indicating prolonged presence in lung tissues. Compared to drug dispersion, the mannosylated-drug-NLCs showcased a maximum AUC value in the lungs of 2691.83 h µg/mL, representing a twofold increase in bioavailability [75].

### 5.4. Liposomes

Liposomes have become a potential drug delivery vehicle because they have a variety of properties, including the ability to encapsulate high doses, to deliver hydrophilic and hydrophobic drugs, to extend the circulation time of the drug, to generate low adverse effects, to control drug delivery, to increase rate of dissolution, and to target drugs to specific cells; biocompatibility; biodegradability; ease of manufacturing; and versatility [56]. Phospholipids and sterols are common components of liposomal substances, which provide the vesicles with stability and unique properties [11]. These qualities make liposomes an appealing choice for delivering a range of antimycobacterial medicines. Liposomes are frequently used to treat bacterial infections because they stop drug breakdown, provide a controlled drug release, and, in some circumstances, allow medication transport through the bacterial membrane to the intracellular milieu [76]. Additionally, liposomes can provide a promising delivery method for negatively charged oligonucleotides since these molecules can electrostatically interact with cationic liposomes to form complexes [77].

In addition to conventional drug delivery, new potential for direct nasal administration of anti-TB drugs to the lungs has been made possible by nanodrug delivery systems based on liposomes. This technique has the advantage of delivering drug concentrations that are pharmacologically effective in alveolar macrophages, producing better therapeutic outcomes [78].

Rinaldi et al. [79] prepared rifampicin-laden liposomes to target *Mycobacterium abscessus*, using anionic DPPG and HSPC via the thin-film hydration technique. These rifampicin-laden liposomes exhibited a PDI and zeta potential of 116.7 ± 9 nm, 0.20 ± 0.01, and −41.7 ± 2.0 mV, respectively. In vitro drug release from these liposomes was approximately 100% within 24 h. The biological activity was evaluated by comparing three groups: untreated macrophages infected with *M. abscessus*, infected macrophages treated with 24, 48, or 96 µM concentrations of the plain drug, and infected macrophages treated with 24, 48, or 96 µM of the rifampicin-laden liposomes. Both the plain drug and the rifampicin-laden liposomes effectively reduced mycobacterium viability, but the latter showed a significantly greater effect. The liposomes containing rifampicin showed no toxic effects on macrophages. The rifampicin liposome formulation could enhance the drug’s efficacy against intracellular mycobacteria [79].

Nkanga et al. [80] formulated liposomes encapsulating isoniazid, using crude soybean lecithin via the film hydration technique for TB treatment. The formulation exhibited a PDI, zeta potential, and entrapment efficiency of 813.00 ± 9.21 nm, 0.29 ± 0.06, −42.80 ± 4.31 mV, and 78.78 ± 2.45%, respectively. The average particle size and zeta potential suggested potential for uptake by macrophages and efficient deep lung deposition. In vitro drug release from the isoniazid-loaded liposomes demonstrated an initial burst release of 35% in the first 30 min, followed by a reduced release of 50% after 12 h. In contrast, the plain drug showed a release of 58% in the first 30 min, followed by a complete release within the subsequent 1.5 h. This highlighted the controlled or sustained release characteristics of the drug-loaded liposomes. These findings suggest the potential for encapsulating anti-TB drugs in cost-effective and readily available crude soybean lecithin liposomes [80].

Despite various benefits of liposomes, such as their safety and biocompatibility, their primary disadvantage as nanocarriers is their instability in plasma. Selective serum proteins (opsonins) bind to the surface of liposomes when they enter the bloodstream, indicating their presence. The mononuclear phagocyte system (MPS), which seizes liposomes and expels them from the bloodstream, recognizes this signal. Although the opposite has also been recorded, in general, larger liposomes are cleared from blood circulation more quickly than smaller ones, and negatively charged liposomes have a shorter half-life in the bloodstream than neutral liposomes [11]. However, it can be combated using functionalization with polyethylene glycol (PEG) and various kinds of ligands such as antibodies and other molecules which increase specificity to the infected site.

### 5.5. Nanoemulsions (NEs)

Nano-emulsions are thought to be one of the most promising options for increasing the oral bioavailability of anti-TB medicines to increase their therapeutic efficacy. A nano-emulsion loaded with anti-TB medicines can quickly overcome biological barriers to enter systemic circulation and, as a result, achieve the target for lowering the load of *M. tuberculosis*. Additionally, the lipidic composition of such systems makes it easier to target the medications to the lymph nodes, improving drug absorption and reducing the frequency of administration [12,81]. In addition, based on the possession of special characteristics such as physical stability, increased surface area, prolonged circulation time, amphiphilicity, specific drug targeting, tumor imaging properties, optical clarity, biodegradability, improved aqueous solubility, and bioavailability, researchers’ focus has shifted to nano-emulsions [82,83]. Additionally, nano-emulsions can have their surfaces altered to permit both passive and active drug targeting [83].

Hussain et al. [84] delivered rifampicin transdermally using a cationic nano-emulsion gel formulated with capmul, labrasol, and acconon to exert antimycobacterium potential. The nano-emulsion was produced using the spontaneous titration method, and carbopol gel was utilized to transform the optimized nano-emulsion into a nano-emulgel. The nano-emulsion labeled as CNE-IV, with an S_max_ ratio of 3:1, was identified as the optimal formulation. It had a mean droplet size, PDI, zeta potential, viscosity, and pH of 89.8 ± 11.4 nm, 0.12, +32.81 mV, 35.4 ± 1.4 cps, and 7.4, respectively. It was observed that the optimized cationic nano-emulsion (OCNE-1) released 57.54 ± 2.9% of the drug within 4 h. In contrast, the OCNE-1-based gel (OCNE-1 gel) and OCNE gel containing transcutol HP (OCNE-IT gel) released only 19.04 ±1.5% and 32.97 ± 1.7%, respectively, of the drug in the same time frame. This difference in release can be attributed to the higher viscosity of the nano-emulgels compared to the nano-emulsion. The enhanced drug release from OCNE-IT compared to OCNE-I is likely due to the presence of transcutol P in OCNE-IT. When compared to the OCNE-I nano-emulsion, the drug solution, and OCNE-I, the OCNE-IT gel showed increased permeation flux (51.32 ± 0.5 µg/cm^2^ h), permeation coefficient (2.566 ± 0.08 cm/h), drug deposition (994.404 µg/cm^2^), and enhancement ratio (7.16) values. The C_max_, T_max_, elimination half-life, and volume of distribution (Vd) for the OCNE-IT formulation were 27,900 ng/mL, 6.0 h, 5.09 h, and 0.226 L, respectively, compared to 5890 ng/mL, 2.0 h, 2.03 h, and 0.382 L for the drug administered orally. The AUC after transdermal application was 328.2 µg h/mL for the OCNE-IT gel, whereas it was 76.31 µg h/mL for orally administered rifampicin [84].

### 5.6. Polymeric Micelles (PMs)

Due to their promising outcomes, polymeric micelles are frequently used to deliver anti-TB medicines. These formulations are known to make poorly soluble drugs more soluble and increase bioavailability, stability, extended circulation, and controlled release while simultaneously reducing toxicity, antigenicity, and immunogenicity with improved tractability [85]. This versatile nanocarrier enables the delivery of anti-TB drugs via the oral, ophthalmic, parenteral, and intranasal routes to accomplish the site-specific delivery of the drugs. The ability to maintain a steady concentration for an extended period of time is made possible by the sustained release of the drug from the miceller structure. Their nanometric size range of 10–200 nm allows them to pass through blood capillaries without being detected by the reticuloendothelial system (RES) while also preventing premature excretion via glomerular filtration [86]. Additionally, these structures enabled quick drug penetration across the cell membrane, either by enhancing drug uptake via miceller structures filled with the drug or by using an alternative internalization pathway (endosomes) [87]. Due to the precise delivery of the therapeutics at the affected site, we can thus achieve longer mean residence time of the therapeutics in systemic circulation accompanied by an altered pharmacokinetics profile, reduction of required dose of administration, improved bioavailability of the drug, and decreased unfavorable toxicities [88]. These unique structures also provide benefits in improvement of drug delivery in which the hydrophobic core can accommodate lipophilic drugs whilst the hydrophobic exterior can be modified with other polymers such as PEG to improve the circulation time and enable passive and active drug targeting [89].

Praphakar et al. [90] fabricated PMs containing rifampicin and isoniazid for the effective treatment of TB. The process for creating the polymeric core involved several steps: initially, chitosan (CS) was combined with polycaprolactone (PCL) to produce CS-g-PCL (given that polycaprolactone is frequently used in drug delivery for numerous bioactive molecules). Subsequently, an amide bond was formed with maleic anhydride-isoniazid (MA-INH). Lastly, CS-g-PCL was joined with the MA-INH component to produce the CS-g-PCL/MA-INH polymeric core. Rifampicin (RF), another anti-TB drug, was loaded onto CS-g-PCL/MA-INH using dialysis, resulting in the formation of RF-CS-g-PCL/MA-INH PMs. The particle sizes of the PMs were recorded as 183.4 and 211.56 nm, respectively. The zeta potential for CS-g-PCL/MA-INH and RF-CS-g-PCL/MA-INH was measured at 11.2 and 26.3 mV, respectively. Transmission electron microscopy revealed a higher dispersion without aggregation in both CS-g-PCL/MA-INH and RF-CS-g-PCL/MA-INH PMs, indicating the stability of the polymeric micelles. In vitro drug release of isoniazid and rifampicin from RF-CS-g-PCL/MA-INH was 85.19% and 75.78% respectively at pH 5.5 on the 12th day, while it was 86.73% and 79.32% on the 24th day at the same pH. This suggests a pH-dependent release pattern for RF-CS-g-PCL/MA-INH, which is ideal for a macrophage environment. The minimum inhibitory concentrations for isoniazid, rifampicin, CS-g-PCL/MA-INH, and RF-CS-g-PCL/MA-INH PMs were determined to be 25.89 ± 0.9, >100, 13 ± 1, and 4.87 ± 0.49, respectively, showcasing the superior potential of PMs over standalone rifampicin and isoniazid drugs. The anti-TB efficacy was evaluated using the luciferase reporter phage method, and decreasing RLU levels indicated the suppression of *M. tuberculosis* H37Rv growth by RF-CS-g-PCL/MA-INH PMs. The toxicity of RF-CS-g-PCL/MA-INH was assessed in relation to rifampicin, isoniazid, chitosan, CS-g-PCL, and CS-g-PCL/MA-INH against U937 and L929 cells using the MTT assay. The RF-CS-g-PCL/MA-INH micelles demonstrated superior inhibitory action on U937 cells, a result attributed to the combination of anti-TB drugs in the micelles. These micelles also had a beneficial effect on L929 cells, suggesting their potential to mitigate the side effects of RF-CS-g-PCL/MA-INH. Therefore, the non-survival of U937 cells and survival of L929 cells when treated with RF-CS-g-PCL/MA-INH micelles revealed the cytotoxic efficacy of the micelles against the disease. Consequently, spherical RF-CS-g-PCL/MA-INH micelles could be considered promising vehicles for controlled and sustained delivery of anti-TB drugs to the macrophage intracellular compartment [90].

### 5.7. Dendrimers

Dendrimers are polybranched, three-dimensional, nanometric, monodispersed, star-shaped vesicles comprising numerous branches on interior surface, a central core, and various functional groups on exterior surface [91]. They have certain structural and chemical characteristics such size (less than 100 nm), shape, and molecular weight. Dendrimers have the potential to prolong drug release, increase the solubility of hydrophobic compounds, and improve the permeability of nanoconjugates across a variety of biological barriers [65]. Although several dendrimers have been applied for drug delivery in various diseases, polyamidoamine (PAMAM) and polypropylene imine (PPI) are extensively used owing to their hydrophilic nature, biocompatibility, and non-immunogenicity [92]. A combination of drugs can be included in dendrimers thanks to the functional groups found on their external surface. It is possible to modify these functional groups to offer drug targeting at the particular site [93]. Additionally, employing dendrimers for drug delivery results in increased drug bioavailability, stability, and water solubility, as well as decreased side effects, larger dose loading, increased medication efficacy, and regulated as well as prolonged drug release [16].

Dendrimers display several advantages as drug carriers, but they also exhibit hemolytic and cytotoxic capabilities, raising serious concerns regarding their safety. Surface functionalization of functional groups found on the external surface of dendrimers can lessen these harmful effects. Polyethene glycols can be used to functionalize the surface of dendrimers, which enhances drug circulation time due to EPR and reduces toxic effects [65].

Ahmed et al. [94] prepared surface-functionalized 4.0 G PAMAM dendrimers for the effective delivery of rifampicin to combat TB. The peripheral coverage of the 4.0 G PAMAM dendrimer using PEG varied between 38% and 100%. Drug-loaded dendrimers with PEG contents of 0%, 38%, 49%, 70%, 85%, and 100% displayed particle sizes of 5 ± 1%, 22 ± 2%, 16 ± 2%, 13 ± 3%, 13 ± 2%, and 21 ± 3% respectively. Their PDI values were 0.31 ± 0.09, 0.34 ± 0.04, 0.35 ± 0.02, 0.39 ± 0.09, 0.28 ± 0.07, and 0.37 ± 0.05 respectively. The entrapment efficiency was recorded as 7.50 ± 1.15%, 72.50 ± 1.64%, 71.25 ± 2.80%, 70.0 ± 3.20%, 65.0 ± 1.77%, and 78.75 ± 0.75% respectively. Dendrimers with no PEG had a drug loading of 26.09 ± 1.35%, and this value increased with a higher PEG concentration, reaching 43.85 ± 1.69% for dendrimers with a 49% PEG concentration. The zeta potential was consistently positive, exceeding 12 mV, and dynamic light scattering (DLS) affirmed the nanoscale nature of the dendrimer formulations. Both differential scanning calorimetry (DSC) and scanning electron microscopy (SEM) supported the drug entrapment and indicated the spherical or semi-spherical shape of the dendrimers. The drug release from the formulation showed an initial burst followed by sustained release. When compared to the unmodified formulation and free medication, PEGylated dendrimers had a more gradual release rate. As the degree of PEGylation increased, the drug release of rifampicin diminished, indicating that PEGylation negatively impacted drug release from the formulation. The dendrimers’ toxicity was assessed using the MTT assay, which showed that PEGylating the dendrimers significantly reduced their toxicity. PEGylated dendrimers were found to be non-toxic to raw cells up to a concentration of 5 µM, maintaining over 80% cell viability. Specifically, PEGylated dendrimers with 85% and 100% PEG exhibited less toxicity to the raw cells compared to the control [94].

### 5.8. Carbon Nanotubes (CNTs)

Researchers have given CNTs a great deal of attention as a potential drug carrier to deliver anti-TB medicines because of a variety of properties including reduced size, increased surface area, high drug loading capability, regulated and sustained release of the pharmaceuticals, and drug targeting [95]. Nanodrug delivery via functionalized CNTs can be used to avoid bacterial multidrug resistance and lower drug dosage. Both single-walled CNTs (SWCNT) and multiwalled CNTs (MWCNT) have been found in studies to be able to thwart the development of drug resistance to several drugs by destroying bacterial cell walls, inducing oxidative stress, and shattering bacterial DNA or macromolecules. Because nanofluids and nanoparticle suspensions have great dispersion stability and bioavailability, they can also be used to construct this CNT system and are hence a feasible drug delivery option [96].

Due to their improved hydrophilicity and lower cytotoxicity, CNTs should be functionalized using various polymers, chemical groups, or biomolecules to assure their targeting ability and safety in the treatment of cancer. By covalent and non-covalently connecting different kinds of polymers and chemical groups to the surface of CNTs, functionalization of the CNTs’ surfaces can be accomplished [65].

Sheikhpour et al. [96] developed a MWCNT nanofluid with conjugated isoniazid and fluoxetine to enhance drug delivery efficacy and combat in vitro drug resistance. The minimum inhibitory concentration (MIC) for the combination of MWCNT with isoniazid (MWCNT-INH) and MWCNT with fluoxetine (MWCNT-FLX) against H37Rv strains, XDR, and MDR was 0.78 + 55.5 (INH 0.067 + FLX 24), 3.125 + 55.5 (INH 0.26 + FLX 24), and 12.5 + 6.93 (INH 1.04 + FLX 3.50), respectively. The fractional inhibitory concentration Index (FIC) for the combination of MWCNT-INH and MWCNT-FLX against H37Rv, XDR, and MDR strains was determined to be 0.5, 2, and 1.5, respectively. It was found that secretion levels of TNFα and IL6 increased in all treated groups, suggesting the potential of drug-entrapped MWCNT to activate the pro-inflammatory pathway in macrophages infected with TB strains due to the efficient elimination of the bacteria. The secretion levels of TNFα and IL6 from macrophages infected with TB were consistent between free MWCNT and drug-entrapped MWCNT, showcasing MWCNT’s potential for targeted delivery to intracellular bacteria. It was noted that the expression of genes, specifically inhA and katG, remained stable under this drug delivery system. Therefore, it is evident that drug conjugation can bolster antibacterial activity against all strains, and both the free and conjugated forms of these medications synergistically boost each other’s effects [96].

### 5.9. Metallic Nanoparticles (MNPs)

MNPs are one of the most effective methods of drug delivery against the contagious *M. tuberculosis*. Due to its bacterial selectivity, reducible size, and extra antibacterial capabilities, this carrier represents a highly promising new carrier for the treatment of TB. When functionalized with targeting ligands that provide controlled deposition into infected cells, MNPs have also been reported to offer improved targeting, gene silencing, and drug delivery. Several MNPs, such as iron oxide nanoparticles (IONPs), zinc oxide nanoparticles (Zn ONPs), copper nanoparticles (Cu NPs), gold nanoparticles (Au NPs), and silver nanoparticles (Ag NPs), are used to treat TB.

Paz et al. [97] encapsulated isoniazid in biocompatible MIL-100 NPs, which were subsequently microencapsulated in mannitol microspheres (Ma MS) containing the iron (III) trimesate metal–organic framework (MOF). This microencapsulation was carried out using the spray-drying technique. The resulting microspheres, obtained in a dry powder form, displayed the desired characteristics, including the appropriate morphology and aerodynamic properties essential for deep lung drug delivery. Moreover, it was discovered that the mannitol microencapsulated MIL-100 NPs loaded with isoniazid (Ma-INH@MIL-100 MS) had a superior drug-loading capacity and demonstrated varied drug release in different aqueous environments. Both Ma-INH@MIL-100 MS and MIL-100 NPs, as well as mannitol, showed minimal toxicity to A549 cells and were efficiently internalized in the cytoplasmic region. Due to their biosafety and tailored pulmonary formulation, these preparations hold significant potential for the local pulmonary treatment of various infectious diseases, including TB [97].

Numerous nanocarriers loaded with anti-TB drugs are summarized in Table 1.

## 6. Delivery of Nanoformulations for TB Treatment

**Figure 4:**
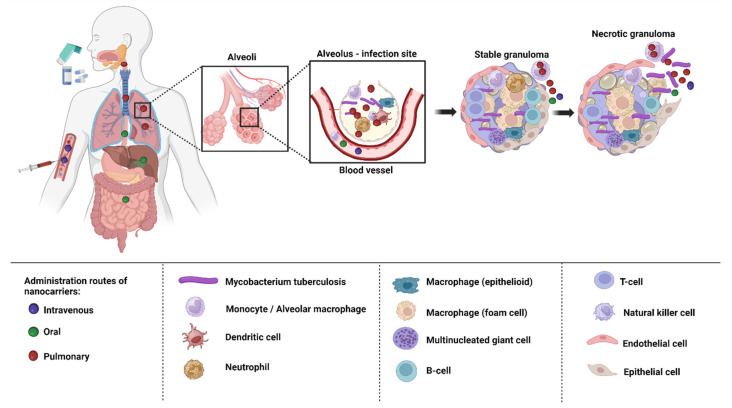
Drug targeting macrophages and granulomas via different routes of administration.

Depending on the biological milieu and obstacles that nanocarriers must overcome, the various ways of administering nanocarriers have varying restrictions. Delivery of nanocarriers via the pulmonary, oral, intravenous, and topical routes may be effective in treating TB. In order to attain the best efficacy, nanocarrier properties can be customized to the intended delivery method [135]. The targeting of infected alveolar macrophages and granulomas employing nanocarriers via various routes of administration has been depicted in the Figure 4.

The oral route is the preferred method of administering nanocarriers because it is noninvasive and more practical for patients to finish their therapy. Low pH and highly proteolytic conditions in the stomach medium, as well as hepatic first-pass metabolism, restrict the range of formulations that are achievable and lower bioavailability [136,137]. Intravenous administration enables the swift absorption of the drugs into systemic circulation and straight into the bloodstream without having to go through first-pass metabolism, which also offers a more accurate control of the dose that is provided [137]. Nanocarriers injected intravenously are either cleared by the MPS or are associated with proteins (protein corona) [138]. Both the oral and intravenous route of administration are associated with adverse effects [139]. The non-invasive topical route enables prolonged release and local activity, resulting in fewer adverse systemic effects, and bypasses hepatic first-pass metabolism. Given that cutaneous TB is a rare illness, it might be advantageous, although little research has been carried out on this approach [140].

The most effective method of treating TB is to directly target the lungs, because they are the primary infected organs in TB. Additionally, compared to the oral route, it enables the achievement of a more effective therapy with lower administration doses and a corresponding reduction in toxicity. Since anti-TB medications have limited potential if administered orally due to their low water solubility, poor biodistribution, and considerable side effects, pulmonary delivery appears promising [141]. The use of the pulmonary route is further encouraged by highly enhanced bioavailability because the activity of the drug-metabolizing enzymes in the organs is less than it is in other organs such as liver and gastrointestinal tract [142]. It can also encourage patient compliance because it is non-invasive and self-administered. However, the structure and function of the respiratory system also play a role in pathogen defense. Consequently, it can be difficult to overcome the biological and structural barriers in the respiratory system [143].

## 7. Biological Barriers to Delivery of Anti-TB Drugs

**Figure 5:**
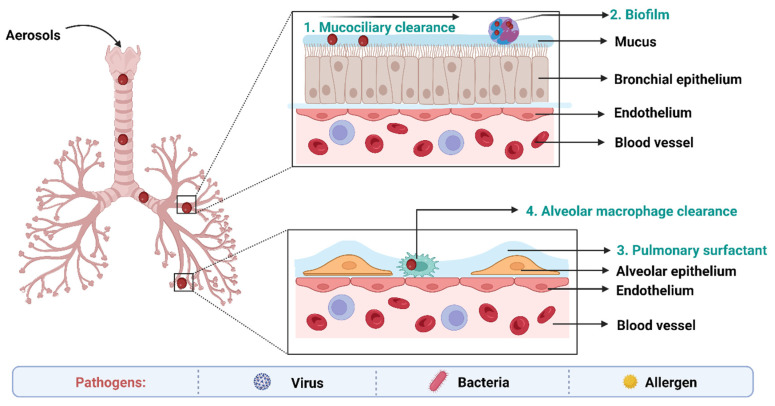
Biological barriers in targeted drug delivery to lungs in TB.

The main obstacles to drug absorption after pulmonary administration for medications administered via inhalation include numerous clearance systems which are exposed to pharmaceuticals. The barriers to delivery of anti-TB drugs to the lungs has been depicted in the Figure 5.

On the epithelium of the trachea and bronchial tree, the mucus layer is present. Its main components are water (90–95%), mucins (primarily MUC5AC and MUC5B), and other substances such as DNA, lipids, electrolytes, proteins, cells, and cell debris (3–8%) [144]. High-viscoelasticity mucus, which is mostly attributed to mucins (high molecular weight (MW) glycoproteins), prevents xenobiotics such inhaled nanoformulations from invading [145]. As a result, nanoformulations and mucus interact in a variety of ways when the former are inhaled into the respiratory system. With a pore size of around 340 nm, the physical barrier will prevent big nanoformulations from reaching the epithelium. Mucus’s negative charge makes it easier for it to interact electrostatically with positively charged nanoformulations, which has an impact on how those nanoformulations move around [146]. Additionally, other sticky interactions between inhaled formulations and mucus, such as hydrophobic contacts and hydrogen bonding interactions, may also result in the retention of nanoformulations. These bio–nano interactions rely mostly on the physicochemical characteristics of nanoformulations. As a result, the greatest barrier to nanoformulations transport in the respiratory system is the bio–nano interaction between nanoformulations and mucus [147].

Pulmonary surfactant (PS), an amphiphilic lipoprotein complex, is secreted by alveolar type II (AT-II) cells in the pulmonary epithelium. PS is made up of surfactant proteins and phospholipids [148]. Surfactant proteins can promote the adhesion and agglutination of certain drugs by mucosal cilia, macrophages, and monocytes. Lung surfactants may also be used to remove therapeutic medications or drug carriers from the body [149].

Numerous immune system-related cells, such as macrophages (MPs), dendritic cells (DCs), and neutrophils, are widely dispersed in the respiratory tract as a result of its innate defense mechanisms. The numerous pathogen-associated molecular pattern receptors on these cells may be able to identify the inhaled medicines and damage-associated molecular pattern receptors [150]. More than 90% of the pulmonary immune cell population is made up of MPs that are found in the bronchial tree and alveolar region, where they work with neutrophils to internalize and digest inhaled medicines [151].

Inhaled nanoformulations initially come into contact with MPs and DCs in the lower respiratory tract, resulting in nanoformulations with smaller size, and are best taken up by MPs, whilst larger one are phagocytosed by DCs [152]. The phagocytosis mechanisms of MPs and DCs are size-dependent and opsonin-dependent. The opsonized nanoformulations are more likely to be recognized by the MPs’ membrane receptors because the opsonin proteins (antibody, complement, and fibrinogen) are quickly adsorbed on the surface of nanoformulations [153]. The identification and phagocytosis of inhaled nanomedicines by MPs play a more significant function than DCs in the lung clearance process because the optimal size of particle deposition in the deeper respiratory tract is larger than 500 nm. Additionally, the pulmonary administration of nanoformulations may result in side effects, including cytotoxicity and inflammatory reactions [145].

Metabolic enzymes in lung epithelial cells also serve as another barrier. Trypsin, antitrypsin, and protease are examples of metabolic enzymes that are present on the surface of bronchial and alveolar epithelial cells as well as pulmonary smooth muscle cells. These enzymes help in the breakdown and metabolism of therapeutic drugs [154].

Biofilms can also act as a significant physical barrier to pulmonary inhalation. A biofilm is a structured community made up of microbial cells that are attached to an inert or biological surface and surrounded by their extracellular matrix, and they serve as a significant barrier to the effective penetration of antimicrobial agents [149].

## 8. Ligand Conjugated Nanoformulations for Circumvention of Pulmonary Barriers

Drug targeting is crucial because it ensures that the therapeutic agent is delivered directly to the site of disease or infection, maximizing its efficacy while minimizing potential side effects elsewhere in the body. Targeted delivery means that a smaller amount of drug can have a more potent effect, reducing the overall dosage and thereby lessening the chances of adverse reactions or drug toxicity.

The target organ or tissue is typically chosen based on the location of the disease or infection. For diseases such as TB, the primary target would be the lungs, where the TB bacteria predominantly reside. However, TB can also affect other organs, and thus, the choice of target would depend on the disease’s manifestation. Scientific research, patient diagnostics, and understanding of disease progression play significant roles in deciding the target.

**Figure 6:**
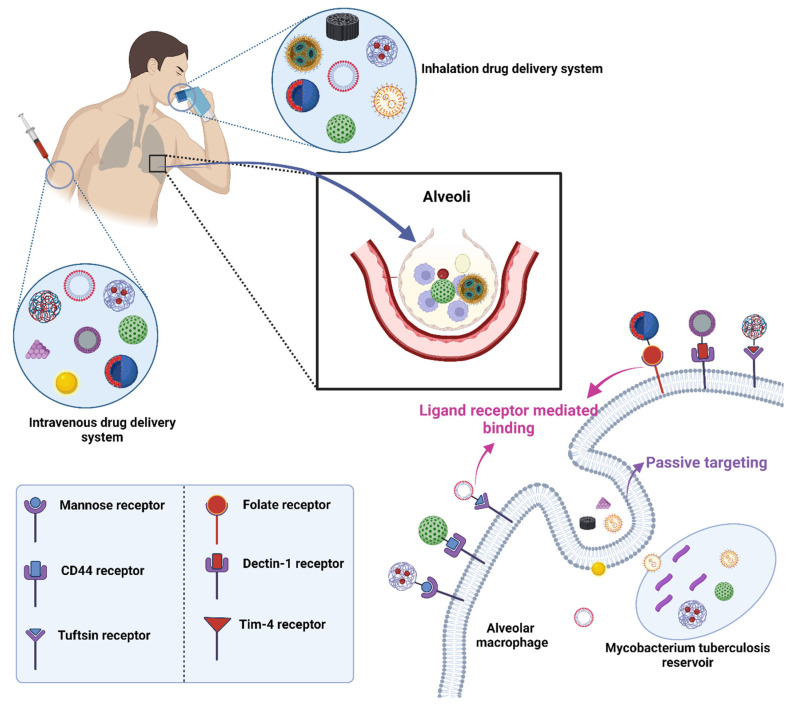
Drug targeting employing conjugation of ligands with drug-loaded nanocarriers in TB.

The mechanism for targeting often involves the use of ligands that can bind specifically to receptors found on the desired target cells. In the context mentioned, nanocarriers are employed as the delivery vehicles. As they move through the body, processes such as opsonization and phagocytosis work to remove them. However, when these nanocarriers are coupled with specific ligands, they can bind to receptors on target cells, such as macrophages, enhancing their ability to deliver drugs directly to the desired location (Figure 6). The choice of ligand is crucial: for instance, mannose, mycolic acid, folate, aptamers, and hyaluronic acid have been used to target TB sites [25]. The environmental conditions, such as pH or the presence of certain enzymes, can also play a role, as some drug delivery systems are designed to release their payload under specific conditions.

### 8.1. Mannose Targeting

A C-type lectin receptor called the mannose receptor (CD206) may detect ligands that include a terminal mannose, *N*-acetylglucosamine, or fucose moiety. Both dendritic cells and the majority of tissue macrophages express this receptor in high amounts [155]. It controls inflammatory signaling pathways as well as endocytosis and phagocytosis. Additionally, it is crucial for the phagocytosis of *M. tuberculosis*, for preventing phagosome-lysosome fusion, and for the intracellular survival of the bacteria [156]. Additionally, the mannose receptor contributes to the development of granulomas. Based on these results, targeting the mannose receptor for TB treatment appears promising, and intracellular co-localization of the bacteria and the nanocarrier is more likely given that they may use the same macrophage entry channel [135]. Numerous studies have concentrated on macrophage targeting via the mannose receptor and shown enhanced absorption of the mannosylated nanocarriers compared to non-targeted formulations [157,158].

Goldoporpora et al. [159] prepared mannosylated polymeric micelles loaded with a combination of rifampicin and curcumin. It was found that addition of mannose resulted in a 5.2-fold improved microbicidal effectiveness of co-loaded drugs against *M. tuberculosis* H37Rv in comparison to their equivalent mannose-free polymeric micelles [159]. Khan M et al. [160] fabricated PNPs loaded with a combination of rifampicin and pentamidine to treat cutaneous leishmanisis and observed that optimized formulation demonstrated superior percent inhibition, more macrophage absorption, and lower IC_50_ value against promastigotes. Additionally, when compared to 0.8% formalin, an in vivo investigation on skin irritation and histopathology showed that the optimized formulation behaved in a safe and non-irritating manner [160].

### 8.2. Folic Acid Targeting

Folic acid is necessary vitamin required for cell growth [161]. This essential molecule is required for DNA replication and repair and RNA synthesis and contributes to the metabolism of amino acids, phospholipids, and nucleotides [162]. Via endocytosis, folic acid derivatives are taken up by cells via the action of foliate receptors. One of the several isoforms of folate receptors, folate receptor α, is overexpressed on the surface of activated macrophages associated with autoimmune and inflammatory illnesses as well as on tumor-associated macrophages (TAMs) [163]. Additionally, several different cancer cell types have elevated folate receptors. Folic acid and its derivatives are therefore commonly employed as effective ligands to target cancer cells, TAMs, and activated macrophages with imaging and therapeutic agents in inflammatory disorders [164]. However, fewer studies have examined the potential of targeting macrophages with folate-functionalized nanocarriers in the treatment of intracellular infections such as TB.

Shah et al. [126] prepared three types of rifampicin-loaded oleic acid-based nano-emulsions, namely, a simple drug-loaded nano-emulsion, a chitosan-conjugated nano-emulsion, and a chitosan–folate-conjugated nano-emulsion, and delivered them using nebulization. All nano-emulsions showed better than 95% aerosol emission and greater than 75% inhalation efficiency. The size and surface tension of nano-emulsions, which are inversely related, were the main factors controlling the aerosol output, aerosolized, and inhaled fine particle fractions. Chitosan–folate-conjugated nano-emulsion showed a higher cell internalization capacity, a lower plasma drug concentration, and a larger lung drug content. The nano-emulsions were confirmed to be safe [126].

### 8.3. Hyaluronic Acid (HA) Targeting

Hyaluronic acid (also known as hyaluronan, or HA) is a glycosaminoglycan made up of repeating *N*-acetyl-D-glucosamine and d-glucuronic acid disaccharide units [165]. A significant part of the extracellular and pericellular matrix (ECM), HA is found in tissues throughout the body and, when in contact with immune cells, can signal whether an area is healthy or inflamed. In healthy tissues, high molecular weight HA (>1000 kDa) predominates and exhibits immunosuppressive and anti-inflammatory properties, whereas low molecular weight HA (500 kDa) forms in response to tissue damage or infection as the components of the ECM break down and exhibits immunostimulatory and pro-inflammatory properties [166].

Activated immune cells increase the expression of CD44, the HA binding receptor, during an inflammatory response. The macrophage protein CD44 is abundantly expressed, and AMs can bind HA even under homeostatic (non-inflammatory) circumstances. HA is absorbed by macrophages in a CD44-dependent manner, after which it is moved to the lysosomes [167]. Additionally, *M. tuberculosis* can use HA as a carbon source for multiplication, and CD44 serves as a location for the macrophage to bind to *M. tuberculosis* [168]. Several HA binding and HA interacting receptors, including the highly expressed TLR2 and TLR4 on macrophages, were discovered in addition to CD44. Via TLR2 and TLR4, HA fragments cause the production of inflammatory genes (chemokine and cytokine expression) in macrophages. Because HA possesses a number of modification sites and is biocompatible and biodegradable, it is a prime choice for usage either directly as a carrier or as a targeted ligand on the surface of nanocarriers. Additionally, tactics against viral disorders such as TB could benefit from the immunomodulatory properties of HA. Because CD44 is also overexpressed on certain cancer cells, HA is being researched as a component of drug delivery systems for both the treatment of infections and cancer.

Mukhtar M et al. prepared dry powder of mannosylated chitosan/hyaluronic acid entrapped with isoniazid and found that pulmonary administration was followed by greater deposition of the nano dry powders in the deeper region of the lungs. The combination of mannose-anchored chitosan and HA preserved and enhanced the ability of NPs to interact with macrophages [169]. Indeed, nanocomposite powders possess physical chemical properties that makes them highly versatility for pulmonary drug delivery [170,171].

### 8.4. Tuftsin Receptor Targeting

Tuftsin is a naturally occurring tetrapeptide that is created via the enzymatic cleavage of the CH2 domain of IgG. Tuftsin is harmless to humans and animals and has anticancer, chemotaxis, and phagocytosis-stimulating activities. By means of receptor-mediated endocytosis, tuftsin is taken up by macrophages and polymorphonuclear phagocytes. Tuftsin and its derivatives have been the subject of countless studies over the past few decades because of the broad range of biological actions they have [172]. It was mentioned that neuropilin-1, which is present in most tissues and has, among others, angiogenesis and axonal guidance functions, binds to tuftsin as well [173].

Horvath L et al. [172] conjugated N-substituted derivatives of 4-aminosalicylic acid (a novel antimycobacetrial agent) with tufstin peptides via oxime or amide bonds. These delivery peptides can specifically target cells that express tuftsin and neuropilin receptors, including macrophages and several other cells derived from the lungs. It was shown that the peptide conjugates maintained the 4-aminosalicylic derivatives’ in vitro antimycobacterial activity against *M. tuberculosis* H37Rv. Free medications had little effect on infected cells, while conjugates had activity against intracellular bacteria and had selectivity for different host cell types. The carrier peptides’ intracellular distribution was examined, and it was discovered that the peptides internalize and manifest mostly in the cytosol in a concentration-dependent way. Using Transwell-inserts and spheroids, the penetration capacity of the most promising carrier peptide, OT5, was assessed. The penetration of the pentapeptide through the non-contact monolayers was time- and concentration-dependent [172].

### 8.5. Mycolic Acid Targeting

Numerous substances found in the cell walls of mycobacteria could be used as targeted ligands. Due to their dominance as lipids in the outer cell wall envelope of mycobacterium species, mycolic acids (MAs) are among them and are hence attractive possibilities [174]. It has been demonstrated that the different MA subtypes significantly influence the pathogen’s pathogenicity. Interesting biological processes carried out by MA from *M. tuberculsis* include foam cell production and immunological steering towards Th1 cellular responses, as well as cholestroid-such as characteristics. MA was found to have immunogenic properties. Human CD4 and CD8 (double negative) T-lymphocyte proliferation takes place when CD1b molecules on dendritic cells are exposed [175].

However, little research has been performed on MA-targeted nanocarriers in treatment of TB but the proposed mechanism for MA targeted nanocarriers is that the MA on the NPs’ outer surface may interact with anti-MA antibodies in the area of the infection sites to create a localized immunological complex that could improve the uptake of the NPs by both the infected and nearby uninfected macrophages [176]. Through its attraction to cholesterol, MA may also target the cholesterol found in the plasma membrane of healthy or diseased macrophages and, more intriguingly, in the membrane of phagosomes that contain pathogenic mycobacteria [177,178].

Lemmer et al. [175] reported isoniazid encapsulated PLGA NPs functionalized with MA for efficacious treatment of TB. Using MA as a targeting ligand, encapsulated isoniazid-PLGA nanoparticles (NPs) were administered to bone marrow-derived mouse macrophages that were either uninfected or infected with different mycobacterial strains (*Mycobacterium avium*, *Mycobacterium bovis*, or *M. tuberculosis*). Electron microscopy was used to keep an eye on the NPs’ fate. The findings revealed that whether MA had been added or not, NPs containing phagosomes were quickly processed into phagolysosomes, NPs containing phagolysosomes did not fuse with non-matured mycobacterium containing phagosomes, and fusion events with mycobacterium-containing phagolysosomes were clearly visible, whereas nanoparticle-containing phagolysosomes did not fuse with non-matured mycobacterium-containing phagosomes [175]. Various ligand conjugated nanocarriers for treatment of TB has been summarized in Table 2.

## 9. Patents

The applicability of the nanoparticle-based formulations was further shown by effectively treating mice with *M. tuberculosis* beneath the skin. Once subcutaneously injected, PLG nanoparticles containing rifampicin, isoniazid, and pyrazinamide maintained therapeutic drug levels for 32 days in plasma and 36 days in the lungs and spleen. Additionally, this single subcutaneous injection sterilized the infected mice’s lungs and spleen (36 days following therapy), indicating a greater degree of chemotherapeutic efficiency than daily treatment with free medications (35 oral doses). The nanoparticles at the injection site may form a depot that delivers drugs into circulation gradually, according to the scientists’ hypotheses [197].

Balaji Narasimhan and Bryan Bellaire (2010) submitted a patent (US8449916B1) which offers compositions and procedures for treating microbial infections in animals, preventing the growth of microorganisms within infected cells, and eliminating pathogens inside infected cells. An animal in need of such therapy may be given an effective antimicrobial dose of a composition made up of polyanhydride microparticles or nanoparticles that are designed to include a variety of antimicrobial agents. The polyanhydride microparticles or nanoparticles can, for instance, be copolymers of 1,6-bis-(p-carboxy phenoxy) hexane (CPH) anhydride, and sebacic anhydride (SA), 1,8-bis (carboxy phenoxy)-3,6-dioxolane (CPTEG) anhydrides, or various combinations of these. The microparticles or nanoparticles can build up in infected dendritic cells, monocytes, both, or on or in other infected cells.

Over time, surface erosion causes the particles to break down and release antimicrobial agents which kill or inhibit the microbes and treat the infection. These approaches and formulations for treating diseases brought on by infectious agents, notably TB, were described by Protopopova et al. in 2012. For the treatment of infectious disorders, substituted ethylene diamine-containing techniques and formulations are offered. These techniques and substances are used in one embodiment to treat mycobacterial infections, including but not limited to TB. The current invention, according to certain embodiments, includes compositions that include new substituted ethylene diamine compounds together with antitubercular medications including rifampicin, isoniazid, pyrazinamide, and ethambutol. Table 3 summarizes the various patent studies using various formulations for anti-TB drugs.

## 10. Conclusions

TB is a leading cause of death from infectious diseases worldwide. While various drug treatments exist to combat this disease, significant challenges remain, including poor patient compliance, a heavy pill burden, and notably, the development of MDR and XDR in TB patients. Additionally, physiological and pathophysiological barriers in the pulmonary region can hinder the efficient delivery of anti-TB drugs to the target site. The advent of drug nanocarriers offers hope for a more effective TB treatment, potentially reducing MDR and XDR. Nanotechnology-based drug formulations, including PNPs, SLNs, NLCs, NEs, PMs, CNTs, MNPs, liposomes, and dendrimers, offer several advantages over traditional treatments. These benefits include a reduced drug dose, fewer adverse effects, enhanced drug solubility and bioavailability, better patient compliance, and decreased drug resistance, all contributing to faster patient recovery from TB. While these nanoformulations can be administered through various methods, such as orally, intravenously, topically, or via the pulmonary route—each with its pros and cons—the most effective treatment for TB involves the direct delivery of anti-TB drugs to the lungs, the primary organ affected by TB. This direct approach offers several advantages over other methods of administration, including reduced doses, decreased frequency of dosing, and fewer side effects. However, barriers such as mucociliary clearance, biofilm formation, pulmonary surfactants, and alveolar macrophage clearance in the respiratory system challenge direct drug delivery to the lungs. Overcoming these obstacles is essential for efficient drug delivery. Conjugating drug-loaded nanocarriers with specific ligands, such as mannose, mycolic acid, folic acid, HA, and aptamers, allows these emerging therapeutic options to navigate these biological and structural barriers in the respiratory system. This targeted approach improves drug delivery, enhancing TB treatment using nanotechnology. In conclusion, TB treatment can benefit significantly from ligand-functionalized nanoformulations of anti-TB drugs. Nevertheless, despite advancements in TB treatment through nanotechnology-based methods, there’s a pressing need for novel drug therapies for more effective TB treatments, aiming to reduce TB cases worldwide.

## Figures and Tables

**Table 1:** Summary of nanocarriers loaded with anti-TB drugs. SLN: solid lipid nanoparticles; NLC: nanostructured lipid carriers; AUC: area under the curve; TGF-1: transforming growth factor 1; THP-1: Tohoku Hospital Pediatrics-1; siTGF-1: transforming growth factor-β1 siRNA; MRSA: methicillin-resistant *Staphylococcus aureus*; PLGA: Poly(Lactic-co-Glycolic Acid); NPs: nanoparticles; HPMA: N-(2-HydroxyPropyl)MethAcrylamide; PEG: Poly(Ethylene Glycol); CFU: colony forming unit; PAMAM: Poly(amidoamine); NE: nano-emulsion; PMs: polymeric micelles; MWCNT: multiwalled carbon nanotubes; CNTs: carbon nanotubes; MNPs: metallic nanoparticles.

Type of Nanocarrier	Active Drug	Method of Preparation	Key Findings	Reference
SLNs	Rifampicin	O/W modified micro-emulsion followed by high-pressure homogenization	Provided better gastric stability which could contribute to bioavailability.	[98]
SLNs	Ethambutol	Hot homogenization and ultrasonication	Established that dry powder inhalation form of ethambutol-laden SLNs improved efficacy against TB.	[99]
SLNs	Rifampicin, isoniazid, pyrazinamide	Micro-emulsion technique	Established double the growth prevention of standard anti-TB drugs against *M. marinum*.	[67]
NLCs	Ethambutol	Hot homogenization followed by ultrasonication	Displayed improved properties on basis of in vitro evaluation testing.	[100]
NLCs	Isoniazid		Exhibited prolonged drug release over 24 h.	[74]
NLCs	Linezolid	Spray-drying	Provided sustained drug release, mucus penetrability, possible safety at therapeutic doses, in vitro and in vivo macrophage targetability, and preferential deposition in the deep lung.	[101]
Colloidosomes	Pyrazinamide	In situ gelation	Provided improved drug plasma concentration and AUC.	[102]
Chitosan coated SLNs	Rifampicin	Ultrasonication	Chitosan-coated SLNs exhibited greater in vitro muco-adhesive characteristics and greater permeability in alveolar epithelial cells A549.	[103]
Liposomes	Isoniazid	Film hydration method	Provided pH-reliant drug release having greater release in acidic environment.	[104]
Liposomes	Isoniazid	Film hydration method	Provided improved entrapment efficiency, particle size, sustained drug release along with reduction in dosing frequency.	[80]
Liposomes	Rifampicin	Freeze-drying	Suggested improved delivery to macrophages than plain drug.	[105]
Liposomes	Pyrazinamide	Film hydration method	Exhibited significant reduction in bacterial counts in lungs.	[106]
Nanoliposomes	Isoniazid, rifampicin, pyrazinamide	Film hydration method	TGF-1 in human macrophages generated from THP-1 could be dramatically downregulated in vitro by nanoliposomal siTGF-1.	[107]
Hybrid nanoparticles	Linezolid	Nanoprecipitation method	Exhibited improved intracellular and anti-biofilm activities of nanoparticles which was mainly due to extensive build-up of nanoparticles inside the MRSA-infected biofilms and osteoblasts.	[108]
PLGA NPs	Rifapentine	Homogenization followed by solvent evaporation	Exhibited improved pharmacokinetic parameters as compared to free drug.	[109]
HPMA-PLGA based NPs	Rifampicin	Modified nanoprecipitation	Exhibited 4 times more efficacy as compared to free drug against *M. tuberculosis*.	[110]
PLGA NPs	Isoniazid and moxifloxacin	Single emulsion technique	Exhibited better activity in conjugation than individual pure drug.	[111]
PLGA-PEG-PLGA NPs	Isoniazid	Sonication followed by double emulsification	Provided 28 times greater bioavailability as compared to free drug.	[112]
PLGA NPs	Rifampicin, isoniazid	Single emulsion solvent evaporation	Exhibited improved inhibition of *M. tuberculosis* compared to pure drugs alone and drugs in conjugation.	[113]
Alginate modified PLGA NPs	Amikacin, moxifloxacin	Double emulsification	Showed a greater reduction in the number of viable bacteria when compared to formulations with just one drug loaded on a nanoparticle and untreated cells.	[114]
PLGA NPs	Pyrazinamide	Double emulsion	Exhibited improved properties on in vitro evaluation and could proceed to in vivo testing.	[115]
PLGA NPs	Linezolid	Modified emulsion solvent evaporation	Exhibited mass median aerodynamic diameter of 3.38 μm along with sustained release for 120 h in simulated lung fluid.	[116]
PLA-PEG NPs	Linezolid	Nanoprecipitation	Exhibited better activity against a group of Gram-positive bacteria responsible for human infections.	[117]
PLGA NPs	Ethionamide, moxifloxacin, econozole	Nanoprecipitation	Showed that NPs of all three drugs collectively caused reduction in CFUs in lungs as well as spleen.	[118]
PLGA-PEG based copolymer NPs	Rifampicin, isoniazid, pyrazinamide	Double emulsification	Exhibited more sustained release of the drug than free drug, a good indication of potential for effective treatment.	[119]
PLGA NPs	Rifampicin, ofloxacin, ethambutol	Spray-drying	Provided better antimicrobial efficacy on in vitro analysis along with significant synergistic effect for isoniazid vulnerable species.	[73]
PLGA NPs	Ethionamide	Freeze-drying	Provided improved AUC and prolonged release up to 24 h in lung fluid.	[120]
PLGA NPs	Ethionamide	Solvent evaporation	Demonstrated possession of excellent potential for treatment of TB.	[121]
PEGylated PAMAM dendrimers	Rifampicin	Dissolution solvent evaporation	Exhibited more prolonged drug release than free drug along with negligible toxicity.	[94]
PEGylated 5.0G PAMAM dendrimers	Rifampicin	Dissolution solvent evaporation	Provided prolonged release of drug along with reduced toxicity.	[122]
Metal based G4 dendrimers	Isoniazid	Solvent-free technique	Provided synergistic impact and an 85 µg/mL dose reduction when the activity was tested on *M. tuberculosis* H37Ra (ATCC 25177).	[123]
Cationic NE	Rifampicin	High-pressure homogenization	Modifications using chitosan increased permeation efficacy at diseased site.	[124]
NE	Linezolid	Oil phase titration	Provided lymphatic targeting of drug at the target organ only after 8 h of dose.	[125]
NE	Rifampicin	Spontaneous emulsification	Exhibited improved cell internalization potential and decreased plasma drug concentration along with greater quantities of drug in lungs.	[126]
PMs	Rifampicin		Rifampicin-loaded PMs increased the in vitro drug’s microbicidal activity against *M. tuberculosis*-infected THP-1 macrophages up to 2.5-fold.	[127]
PMs	Rifampicin, isoniazid	Co-solvent/evaporation	Exhibited increased oral bioavailability (up to 3.3 times) of rifampicin compared to free drug in the presence of isoniazid.	[128]
PEGylated PMs	Rifampicin, isoniazid	Freeze-drying	Provided more efficacy against sensitive *M. tuberculosis* strains and found to be less hemolytic.	[129]
PMs	Rifampicin, isoniazid, pyrazinamide	Solvent evaporation method	Provided sustained drug release on in vitro evaluation.	[130]
MWCNTs	Isoniazid	Reflux system	Provided better antimicrobial potential at lower concentrations.	[95]
Chitosan/CNTs NPs	Isoniazid		Exhibited reduced numbers of CD3+ and CD4+ T cells in isoniazid/chitosan/carbon nanotube group.	[131]
MNPs	Rifampicin		Loaded drug appeared to be more biocompatible and had stronger antimycobacterial properties.	[132]
MNPs	Ag, ZnO, and Ag-ZnO	Chemical reduction and chemical synthesis	Provided improved efficacy to treat multidrug-resistant and extensively resistant *M. tuberculosis*.	[133]
MNPs	Rifampicin	Green synthesis	Provided a decrease of antibiotic dosage and inhibition of its adverse effects.	[134]

**Table 2:** Ligand-conjugated nanocarriers for treatment of TB. NLC: nanostructured lipid carriers; SLN: solid lipid nanoparticles; NPs: nanoparticles; BCG: bacille Calmette–Guérin; PMs: polymeric micelles; PLGA: Poly(Lactic-co-Glycolic Acid).

Nanocarrier	Ligand	Drug	Result Outcomes	Ref.
NLCs	Mannose	Isoniazid	Exhibited a prolonged residence time in the pulmonary region with higher pharmacokinetics than non-functionalized formulation demonstrating the improved therapeutic efficiency of the mannose functionalized NLC formulation.	[157]
Cationic NLCs	Mannose	Rifampicin	Demonstrated considerably better absorption efficiency in NR8383 cells and alveolar macrophages than unmodified NLCs in cell-specific targeting.	[179]
NLCs	Tuftsin	Rifampicin	Macrophages substantially more frequently internalized tuftsin-containing nanoparticles than tuftsin-free ones. In comparison to free rifampicin, both nanoparticles were twice as efficient against *M. tuberculosis*.	[180]
SLNs	Mannose	Rifampicin	Surface mannosylation accelerated macrophage phagocytosis, showing evidence of an active targeting promotion.	[181]
SLNs	Mannose	Rifampicin	The mannosylation of SLNs increased their internalization in macrophages and confirmed their biocompatibility.	[158]
SLNs	Mannose	Rifabutin	Revealed a nearly six-fold increase in uptake in alveolar macrophages in comparison to uncoated formulation.	[182]
NPs	Mannosylated and Pegylated graphene oxide	Rifampicin	Provided enhanced intracellular rifampicin delivery and pharmacokinetics dramatically improved the effectiveness of rifampicin-driven killing of intracellular BCG and *M. tuberculosis* bacilli in infected macrophages both in vitro and ex vivo.	[183]
Liposomes	Mannose	Isoniazid, rifampicin	Mannosylated liposomes had the strongest anti-TB activity when tested in Balb/C mice. The biodistribution experiments also showed increased drug concentration (accumulation) that was sustained over an extended period of time.	[184]
Gelatin NPs	Mannose	Linezolid	Provided reduction in the dose, frequency of administration, and side effects, resulting in increased patient compliance.	[185]
Gelatin NPs	Mannose	Isoniazid	Intravenous treatment of formulation significantly reduced bacterial numbers in the lungs and spleen as well as the drug’s hepatotoxicity.	[186]
Chitosan NPs	Mannose	Rifampicin	The drug release from conjugated nanoparticles included in in situ gel was determined to be roughly 70.3% at the end of 40 h in simulated synovial fluid.	[187]
Chitosan NPs	Mannose	Clofazimine	Indicated that mannosylated NPs internalized more quickly. Additionally, the H37Rv strain luciferase reporter phage (LRP) experiment demonstrated that clofazimine nanoparticles had 49.5 times greater inhibition and antimycobacterial activity than free clofazimine.	[188]
N,N,N-trimethyl chitosan nanoparticles	Mannose	Etofylline	Provided that therapeutic efficacy of etofylline has significantly improved according to in vivo pharmacokinetic investigations in the Wistar rat model.	[189]
Polymeric micelles	Mannose	Curcumin, rifampicin	Resulted in a huge (5.2-fold) improvement in the microbicidal effectiveness of co-loaded systems against *M. tuberculosis* H37Rv in comparison to their equivalent mannose free polymeric micelles	[159]
Microparticles	Sodium hyaluronate	Rifampicin, isoniazid and verapamil	Ex vivo macrophage infection studies using susceptible and drug-resistant strains were performed, as well as in vitro antimicrobial activity tests. When the powder, which did not affect Ams viability after a five-day exposure, was contrasted with the same formulation without verapamil, no appreciable differences were found.	[190]
NPs, PMs	Galactomannan	Rifampicin	Revealed that both nanocarriers were taken up by RAW 264.7 murine macrophages. Surface modification of nanocarriers causes a notable rise of the intracellular concentration of the drug.	[191]
Microspheres	Hyaluronic acid	Ofloxacin	Provided increased absorption on RAW 264.7 cells and intratracheally delivered microspheres in Sprague–Dawley rats resulted in larger lung accumulation and lower plasma levels when compared to i.v. and oral administration (OFX solution).	[192]
Microspheres	Sodium hyaluronate		Spray-dried microspheres reduced the viability of A549 cells because of the surfactant component	[193]
PLGA NPs	Tuftsin	Coumaron derivative namely TB515	Demonstrated that adding a Pluronic–Tuftsin conjugate coating to nanoparticles significantly enhanced the internalization rate and intracellular activity of the encapsulated therapeutic candidate against *M. tuberculosis*.	[194]
Liposomes	Tuftsin	Rifampicin	Drug-loaded liposomes were at least 2000 times more effective than the free medication at reducing the load of lung bacilli in Mtb H37Rv-infected Swiss albino mice (i.v. infection) when provided twice weekly for two weeks in i.v. administered liposomes.	[195]
Nano-emulsion	Folate	Rifampicin	Chitosan–folate-conjugated nano-emulsion showed a higher cell internalization capacity, a lower plasma drug concentration, and a larger lung drug content. The nano-emulsions were confirmed to be safe.	[126]
Liquid crystalline NPs	Folate	Rifampicin	Revealed the improved intracellular uptake and reduced cytotoxicity of NPs by alveolar macrophages.	[196]
PLGA NPs	Mycolic acid	Isoniazid	Provided improved enhancement in phagocytic uptake of the NPs.	[175]

**Table 3:** List of patents related to nanoformulations of anti-TB treatment. PLGA: Poly(Lactic-co-Glycolic Acid); PCT: procalcitonin; MDRTB: multidrug-resistant tuberculosis; MRSA: methicillin-resistant *Staphylococcus aureus*; MSSA: meticillin-sensitive *Staphylococcus aureus*.

Patent No.	Formulation/Description
US8927024B1	For example, the copolymers of 1,8-bis-3,6-dioxaoctane anhydrides and 1,6-bis-hexane anhydrides, or any combination of these two, may serve as polyanhydride microparticles or nanoparticles. The nanoparticles or microparticles can enter in infected dendritic cells, monocytes, and other infected cells. Over time, surface erosion causes antimicrobial chemicals to escape from the particles, killing or suppressing the microorganisms and curing the infection.
US20090192173A1	For the treatment of infectious disorders, substituted ethylene diamine-containing techniques and formulations are proposed. The current invention includes compositions that include new substituted ethylene diamine compounds together with antitubercular medications including rifampicin, isoniazid, pyrazinamide, and ethambutol in certain embodiments.
US20100310662A1	The oral drug delivery system includes PLGA nanoparticles with an azole encapsulated therein, PLGA nanoparticles with moxifloxacin encapsulated therein, and PLGA nanoparticles with rifampicin encapsulated therein.
US20170044100A1	The current invention offers brand-new indoleamine substances for treating TB, including drug-resistant *M. tuberculosis*, compositions containing indoleamine, and procedures for combining the indoleamine with other biologically active agents to treat TB in a subject in need of such treatment.
US20050084455A1	The present invention into two anti-TB drugs and a biodegradable polymer for drug delivery in a ratio of approximately 1:2 to 2:1, wherein the anti-tubercular drugs are in the ratio of 1:2 to 2:1. Finally, it pertains to a technique of treating pulmonary TB in a subclinical context as well as a procedure for making the composition.
US20070128124A1	The invention offers systems, procedures, and compositions for giving capreomycin in an aerosolized form to those who need it. Aerosol capreomycin administration may be used to lessen the intensity or length of a TB infection as well as to lessen the infectivity of TB patients. This innovation also allows for the use of capreomycin in the creation of a medication that may be administered to someone who needs it through aerosol.
US8697653B2	According to PCT application publication WO2011027290, the invention is a biodegradable, inhalable microparticle formulation for drug administration that contains a drug and a lipid in a certain ratio. The present invention also covers treating pulmonary TB, MDRTB, MRSA, and MSSA pneumonia in mammals by administering a therapeutically effective amount of the formulation. The invention also involves inhaling or intratracheal instilling a microparticle formulation to an animal who needs it.

## Data Availability

Not applicable.

## Abbreviations

Ag NPs: silver nanoparticles; AT-II: alveolar type II; Au NPs: gold nanoparticles; AUC: area under the curve; BCG: bacille Calmette–Guérin; CFU: colony forming unit; CNTs: carbon nanotubes; CPH: (p-carboxy phenoxy) hexane; CPTEG: 1,8-bis (carboxy phenoxy)-3,6-dioxolane; CS: chitosan; Cu NPs: copper nanoparticles; DCs: dendritic cells; ddPCR: droplet digital polymerase chain reaction; DLS: dynamic light scattering; DSC: differential scanning calorimetry; EE: encapsulation efficiency; HA: hyaluronic acid; MA: mycolic acids; HPMA: N-(2-HydroxyPropyl)MethAcrylamide; IONPs: iron oxide nanoparticles; LAMP: loop-mediated isothermal amplification; Ma MS: mannitol microspheres; MA-INH: maleic anhydride-isoniazid; MALDI-TOF: matrix-assisted laser desorption ionization-time of flight; MDR: Multidrug Resistant; MDRTB: multidrug-resistant tuberculosis; MMAD: mass median aerodynamic diameter; MNPs: metallic nanoparticles; MOF: metal–organic framework; MPs: macrophages (MPs); MPS: mononuclear phagocyte system; MRSA: methicillin-resistant *Staphylococcus aureus*; MSSA: meticillin-sensitive *Staphylococcus aureus*; MTB: *Mycobacterium tuberculosis* complex; MW: molecular weight; MWCNT: multiwalled CNT; MWCNT: multiwalled carbon nanotubes; NEs: nanoemulsions; NK: natural killer; NLC: nanostructured lipid carriers; NPs: nanoparticles; PAMAM: Poly(amidoamine); PCL: Polycaprolactone; PCT: Procalcitonin; PDI: polydispersity index; PEG: Poly(Ethylene Glycol); PLGA: Poly(Lactic-co-Glycolic Acid); PMs: polymeric micelles; PNPs: polymeric nanoparticles; PPI: Polypropylene imine; PS: pulmonary surfactant; RES: reticuloendothelial system; RIF: resistance to rifampin; XDR: extensively drug-resistant; SA: sebacic anhydride; SDGs: sustainable development goals; SEM: scanning electron microscopy; siTGF-1: transforming growth factor-β1 siRNA; SLN: solid lipid nanoparticles; SSM: sputum smear microscopy; SWCNT: single-walled CNT; TAMs: tumor-associated macrophages; TB: tuberculosis; TGF-1: transforming growth factor 1; THP-1: Tohoku Hospital Pediatrics-1; VEGF: vascular endothelial growth factor; WHO: World Health Organization; Zn ONPs: zinc oxide nanoparticles.
